# Association between beta-blocker and statin use and mental health in patients following pulmonary embolism: a prospective cohort study

**DOI:** 10.1186/s40359-026-05187-w

**Published:** 2026-07-16

**Authors:** Daniel Sabljo, Simone Fischer, Thomas M. Berghaus, Jakob Linseisen, Christa Meisinger, Timo Schmitz

**Affiliations:** 1 Institute for Medical Information Processing, Biometry, and Epidemiology – IBE, LMU Munich, Munich, Germany; 2 Pettenkofer School of Public Health, Munich, Germany; 3 Medical Faculty, University of Augsburg, Augsburg, Germany; 4 Department of Cardiology, Respiratory Medicine and Intensive Care, University Hospital Augsburg, Augsburg, Germany; 5 Medical Faculty, Ludwig-Maximilians-University München, Munich, Germany

**Keywords:** Depression, Anxiety, Beta-blockers, Statins, Pulmonary embolism

## Abstract

**Rationale:**

Beta-blockers and statins are important drugs for the prevention of several cardiovascular diseases and are often prescribed in pulmonary embolism (PE) patients suffering from cardiovascular comorbidities. The present observational cohort study aims to investigate the potential association between the use of both beta-blockers and statins and the symptoms of depression and anxiety after PE.

**Methods and results:**

This analysis is based on 538 patients with acute PE who were admitted to the University hospital Augsburg between the years 2017 and 2022 and participated in the ‘Lungenembolie-Augsburg (LEA)’ study. The participants were interviewed during their hospital stay and received postal questionnaires three months after discharge. Symptoms of depression and anxiety were measured using the Hospital Anxiety and Depression Scale (HADS). Multivariable linear regression models were used to evaluate the association between both regular beta-blocker and statin use prior to PE and depression and anxiety (HADS-Scores) three months after PE, respectively. While prior use of beta-blockers was significantly associated with depressiveness (β = 0.87 [0.01–1.74], *p* = 0.046) and anxiety (β = 0.87 [CI: 0.05–1.69], *p* = 0.037), statins did not show a significant association with either depression or anxiety.

**Conclusion:**

This study demonstrates an association between beta-blocker use and mental well-being of PE patients, which physicians must be aware of in the treatment of these patients.

**Supplementary Information:**

The online version contains supplementary material available at 10.1186/s40359-026-05187-w.

## Background

Pulmonary embolism (PE) is a clinical condition characterized by the obstruction of the pulmonary arteries usually caused by blood clots dislodging from deep veins [39]. It is a potentially life-threatening disease with a reported mortality rate of about 12% within the first month after diagnosis [4]. Next to acute complications like hypoxemia, right ventricular failure, pulmonary hypertension, arrhythmias, and even cardiac arrest [24], PE patients often suffers from long-term complications like persisting dyspnea and fatigue, reduced ability to perform everyday tasks, and consequently generally reduced quality of life [17]. As a result, many patients suffer from diminished social participation or even isolation [17], which leads to considerable psychological distress that is also reflected in the elevated prevalence of anxiety and depressive symptoms following the acute event [12, 15, 17].

Among many others, important risk factors for deep vein thrombosis and PE are diabetes mellitus, obesity and smoking [33] and PE patients frequently suffer from a variety of cardiovascular comorbidities such as hypertension, hypercholesterinemia or coronary artery diseases [16, 18]; a circumstance that can further aggravate mental health issues in PE. The high prevalence of cardiovascular comorbidities is the reason that many PE patients are prescribed with beta-blockers and statins [5, 20]. However, the effect of these medications may reach beyond their intended cardiovascular effects. Numerous studies have examined the impact of beta-blockers and statins on the psychological well-being of patients, showing diverse emotional responses. Patients taking beta-blockers reported symptoms such as fatigue, mood swings and increased anxiety, with some also experiencing depressive symptoms [1, 22, 27]. In contrast, evidence regarding medication with statins has been inconsistent. While some studies suggested their potential to enhance mental-health by improving cardiovascular well-being, others found no association [14, 26, 42]. Existing studies have mainly focused on the physical aspects of PE treatment; the psychological consequences such as anxiety and depression on the other hand have been often overlooked despite their significant impact on patients´ well-being [15, 19]. Consequently, current literature remains inconclusive regarding the impact of beta-blocker and statin use on mental health in patients recovering from PE. Beta-blockers and statins are prescribed frequently in PE patients and there is a high overall vulnerability of these patients regarding mental health, so it seems necessary that this gap in scientific knowledge must be addressed. Consequently, the aim of the study was to investigate the association between beta-blocker and statin use and symptoms of depression and anxiety three months after the acute event in a cohort of patients hospitalized with PE. This may contribute to a better understanding of the interplay between beta-blocker or statin use and the occurrence of mental health disorders in patients following PE.

## Methods

### Study design and setting

The present study uses data from the ‘Lungenembolie-Augsburg (LEA)’ cohort, an observational single-center cohort study. The cohort includes a total of 811 participants aged 18 and older with either incident or recurrent PE who were admitted to the University Hospital Augsburg between July 2017 and April 2022 and who completed the baseline survey. Further details regarding the LEA study can be found in the published study protocol [30].

The LEA study was conducted in accordance with the Declaration of Helsinki, with ethical approval granted by the ethics committee of Ludwig-Maximilians-Universität München (Date of approval: 1 August 2017. Reference number: 17–378). Written informed consent was provided by all participants or their legal representatives.

### Patient recruitment and baseline assessment

During the patient’s hospital stay, nurses administered the baseline questionnaire via face-to-face interview to collect key patient data including their demographics, symptoms, diagnostic delays, underlying risk factors (smoking status, alcohol consumption etc.) and existing comorbidities (e.g. cancer, respiratory or cardiac conditions, diabetes, neuromuscular disorders, and sleep-disordered breathing). The study nurses were trained to perform the interview according to a detailed standard operating procedure (SOP). Only after successful certification (conducting the interview in accordance with the SOP guidelines under the supervision of the person responsible for the SOP), study nurses were allowed to conduct actual patient interviews.

Furthermore, the regular intake of medications prior to the PE event was assessed. Patients were asked to complete self-administered questionnaires on subjective health status [EuroQol Visual Analogue Scale (EQ VAS) [7, 10] and mental health [Hospital Anxiety and Depression Scale (HADS)]. Additionally, biological samples, including blood, urine, and stool were taken. Clinical data was collected via chart review of the participants’ medical records.

### Clinical data collection: exposure and covariables

The exposure of the present study was regular usage of beta-blockers or statins prior to the acute event. All medications taken within the last 7 days preceding hospital admission were recorded. The preparations were coded according to the German anatomic therapeutic chemical (ATC) classification. Medications were assigned as ‘statins’ only if the compounds taken were defined by the ATC as C10AA or C10BA, and as ‘beta-blockers’ only if the compounds taken were defined as ATC C07.

The analyses also included the following covariables: diagnoses of prior PE events, diagnosis of depression before the PE event, usage of antidepressants, demographic data such as age, gender (male/female), height, weight, chronic conditions, level of education and health-related behavior such as the smoking status. The level of education was categorized into two groups: Participants with more than 9 years of education were classified as having high education, while those with less than 9 years were classified as having lower education. The variable smoking status was categorized in three subgroups as current smoker, ex-smoker, and never smoker. Prior PE and depression were assessed based on whether a documented diagnosis of PE or depression had been established at any point before the patient’s hospital admission. The use of antidepressant medication was assessed by determining whether the patient had taken such medications within the seven days preceding the PE event. BMI was calculated by dividing body weight in kilograms by height in meters squared (kg/m^2^). The BMI of each person was then categorized as normal (< 25.0 kg/m^2^), overweight (25.0–29.99 kg/m^2^) or obese (≥ 30.0 kg/m^2^). The variable chronic conditions was determined by the presence of either chronic kidney disease or diabetes, or both.

### Follow-up

The participants received postal follow-up questionnaires 3, 6 and 12 months and then yearly after their PE. Data of the first follow-up three months after hospital discharge was used for this analysis. These questionnaires included, among other topics, the current medication in use, health-related quality of life, subjective health status (EQ VAS) and symptoms of depression and anxiety (HADS).

### Hospital Anxiety and Depression Scale (HADS)

The HADS is a self-administrated questionnaire used to identify anxiety and depressive symptoms in physically ill patients. The HADS consists of 14 items, seven each for anxiety (HADS-A) and depression (HADS-D). Each item has a four-point response scale ranging from 0 (absence) to 3 (extreme presence). Each of the subscales for anxiety and depression amount to a maximum total score of 21. Elevated scores reflect greater levels of anxiety and depression. A cut-off score of ≥ 8 points for the presence of depression or anxiety has been shown to be appropriate in previous studies [6].

### Sample size and exclusion criteria

**Fig. 1:**
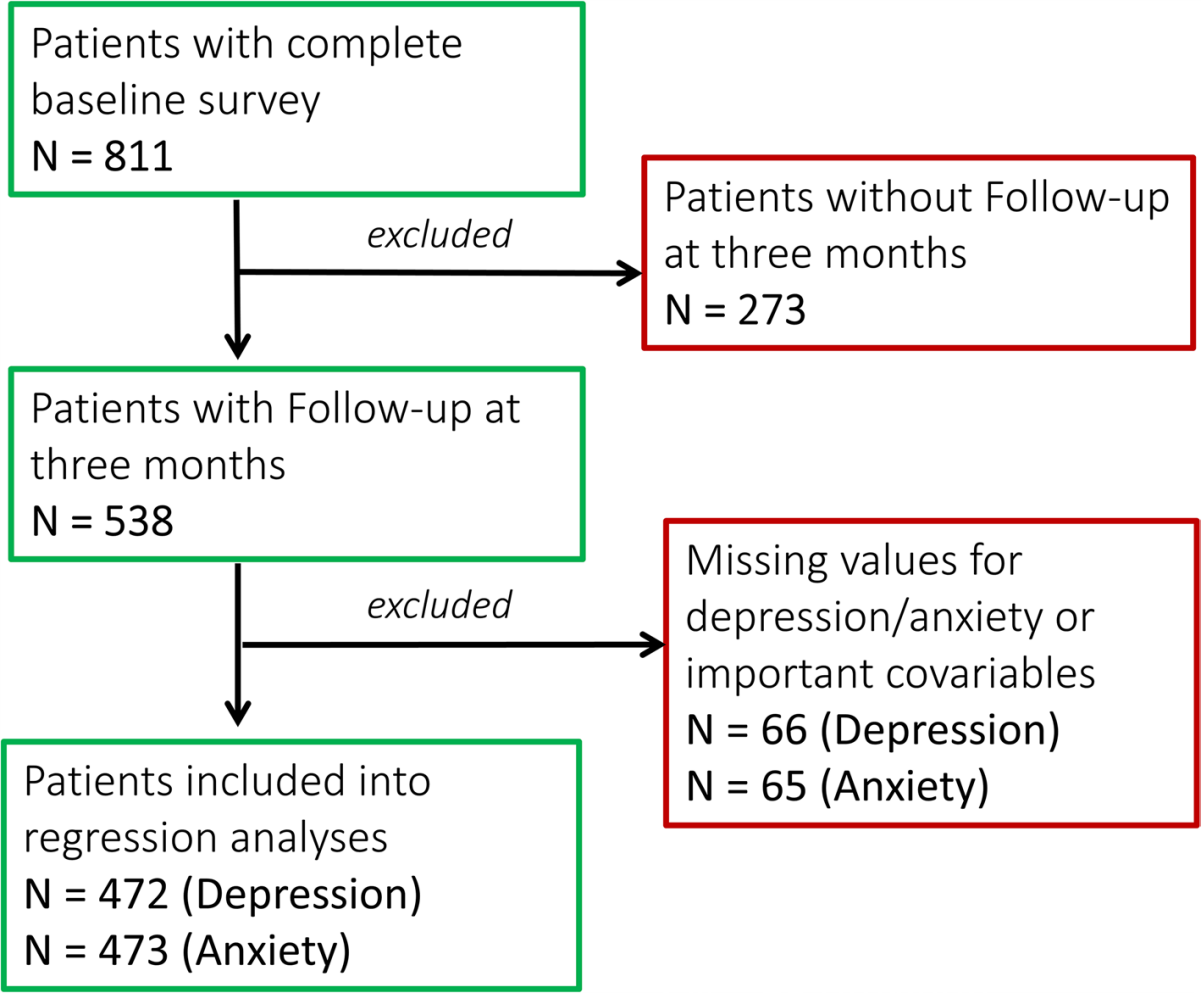
Flow chart displaying all inclusions and exclusions and representing the final sample size

During the recruitment phase a total of 811 patients were admitted to the University Hospital Augsburg and completed the baseline survey. Response rate for the first follow-up three months after the PE event was 66.3% (*n* = 538), see Fig. 1. We examined the missing data patterns for all variables relevant for the regression analysis using R´s VIM package (see supplementary figure S1). As the number of missing values was low and no specific patterns were observed, only patients with valid information on all covariables were included into regression analysis (complete case analysis).

### Statistical analysis

A descriptive analysis of the baseline sample characteristics stratified by depression (HADS-D-Score ≥ 8) and anxiety (HADS-A-Score ≥ 8) after three months was conducted and presented as mean/standard deviation (SD) or median/interquartile range (IQR) for continuous variables and as absolute numbers with percentages for categorical variables. Differences between groups for continuous variables were analyzed via t-test and Mann–Whitney U test, respectively. Chi-square tests were used to examine group differences for categorical variables. Multiple linear regression models were used to examine the associations between the usage of beta-blockers and statins (exposure) and depression and anxiety according to the HADS score (outcome). The objective of the study was not to determine the presence or absence of clinically relevant depression or anxiety, but rather to assess the association across the full range of symptom severity. Therefore, we analyzed the HADS scores as continuous variables. The selection of relevant covariables was based on literature review using PubMed and Google Scholar and on the directed acyclic graph (DAG) method, using DAGitty (Version 3.1). DAGitty is a web-based application for drawing and analyzing causal diagrams. It identifies minimal sufficient adjustment sets for estimating causal effects and detects biasing paths to diagnose insufficient or invalid adjustments [38]. The linear regression models were adjusted for the following covariables: age, gender, depression prior to the PE event, smoking status, medication with antidepressants, prior PE event, body mass index (BMI) and education. We conducted a total of four multivariable linear regression analyses. For each outcome, depression and anxiety, two regression models were calculated: one examining beta-blockers use and the statin medication.

Model assumptions were examined using scatterplots and Shapiro–Wilk tests to confirm the linearity of associations, normality of residuals and homoscedasticity. A sensitivity analysis was conducted to ensure the validity of the results, as the assumption of normality was approaching a critical threshold but remained within acceptable limits. For the sensitivity analysis, a linear regression model examining the association between anxiety and the use of beta-blockers was performed and thereby setting HADS-A scores greater than 15 to 15. This adjustment was made to address a slight right skew in the distribution of the regression residuals caused by a few higher HADS-A values. Variance inflation factor was used to assess multicollinearity.

Furthermore, we performed additional sensitivity analyses: two linear regression models (one for depression and one for anxiety) were calculated using a combined variable for medication (categories: only beta-blockers, only statins, beta-blockers and statins, and none of both [reference group]). The models were adjusted for the same confounders as the main models.

Moreover, to assess if the Covid-19 pandemic has affected the validity of the results, we have performed subgroup analyses. Therefore, the multivariable linear regression models were calculated including either cases before January 1, 2020 (hospitalization and three months follow up before the Covid-19 pandemic) or including only cases hospitalized on January 1, 2020 or after. Finally, we calculated multivariable linear models in the same way as for the main analysis but used intra-hospital HADS values as the outcome.

For all statistical tests, an alpha level of 0.05 was defined. All analyses have been done using R version 4.4.2

## Results

### Sample characteristics

This analysis included 538 participants from the LEA Study who responded to the survey three months post-hospital discharge following a PE event, see Fig. 1. The sociodemographic and clinical baseline information of the included participants, stratified for depression and anxiety three months post-PE, is presented in Table 1. The mean age of the included participants was 63.3 years (SD: 14.5) with 292 male participants (54.3%) and a median BMI of 28.3 (IQR: 24.9—32.9). Notably, 16.8% of the total study population had been diagnosed with depression at some point before hospital admission and 10.8% had experienced a recurrent PE.

**Table 1:** Baseline characteristics of the study population stratified for depression and anxiety three months after pulmonary embolism (PE)

		**Total sample**^**a**^ ***n* = 538**	**Depression**^**b**^ ***n* = 109**	**No Depression** ***n* = 415**	***p* value**	**Anxiety**^**c**^ ***n* = 104**	**No Anxiety** ***n* = 420**	***p* value**
***Age***	Mean (SD*)	63.3 (14.5)	65.8 (12.8)	62.2 (14.8)	**0.013**	63.9 (13.8)	62.8 (14.7)	0.452
***Sex—male***	n (%)	292 (54.3)	65 (59.6)	222 (53.5)	0.299	58 (55.8)	229 (54.5)	0.906
***High school education***	n (%)	152 (28.3)	18 (16.5)	132 (31.8)	**0.002**	16 (15.4)	134 (31.9)	**0.001**
***Married***	n (%)	343 (64.2)	66 (61.7)	268 (64.9)	0.614	64 (62.1)	272 (65.1)	0.658
***Living with a spouse***	n (%)	356 (67.0)	66 (62.3)	281 (68.4)	0.281	67 (65.0)	282 (68.0)	0.656
***HADS***^***d***^*** Depression Score***	Median (IQR**)	3.0 (1.0—7.0)	11.0 (9.0—13.0)	2.0 (1.0—4.0)	** < 0.001**	9.0 (6.0—12.2)	2.0 (1.0—5.0)	** < 0.001**
***HADS***^***d***^*** Anxiety Score***	Median (IQR**)	4.0 (1.0—7.0)	9.0 (6.0—12.0)	3.0 (1.0—5.0)	** < 0.001**	10.0 (9.0—13.0)	3.0 (1.0—5.0)	** < 0.001**
***Bilateral PE***^***g***^	n (%)	409 (77.9)	86 (81.1)	312 (77.0)	0.440	81 (80.2)	316 (77.1)	0.588
***Central thrombus components***^***e***^	n (%)	212 (45.2)	44 (46.8)	163 (44.9)	0.830	36 (40.4)	168 (45.9)	0.419
***Infiltrates***^***e***^	n (%)	192 (41.9)	34 (37.0)	154 (43.5)	0.310	35 (40.7)	155 (43.3)	0.752
***BMI***^***f***^	Median (IQR**)	28.3 (24.9—32.9)	27.8 (25.2—34.3)	28.4 (24.9—32.8)	0.872	28.9 (26.0—35.2)	28.1 (24.8—32.6)	0.207
***Smoking status***					0.902			0.918
*Never smoker*	n (%)	278 (52.4)	57 (53.8)	212 (51.6)		54 (52.9)	214 (51.4)	
*Former smoker*	n (%)	210 (39.5)	40 (37.7)	165 (40.1)		39 (38.2)	168 (40.4)	
*Current smoker*	n (%)	43 (8.1)	9 (8.5)	34 (8.3)		9 (8.8)	34 (8.2)	
***History of depression***	n (%)	90 (16.8)	33 (30.3)	55 (13.3)	** < 0.001**	26 (25.0)	60 (14.3)	**0.013**
***Prior PE***^***g***^	n (%)	58 (10.8)	10 (9.2)	46 (11.1)	0.689	6 (5.8)	48 (11.4)	0.129
***Chronic condition***^***h***^	n (%)	130 (24.3)	38 (34.9)	88 (21.3)	**0.005**	39 (37.9)	85 (20.3)	** < 0.001**
***Cancer***	n (%)	106 (19.7)	25 (22.9)	78 (18.8)	0.405	20 (19.2)	84 (20.0)	0.969
***EQ VAS***^***i***^*** (baseline)***	Median (IQR**)	61.0 (45.0—75.0)	50.0 (35.0—60.0)	66.0 (50.0—80.0)	** < 0.001**	50.0 (40.0—65.5)	65.0 (50.0—80.0)	** < 0.001**
***SARS-CoV-2***^***j***^*** vaccination before baseline***					**0.040**			0.080
*Missing information*	n (%)	102 (18.9)	30 (27.5)	66 (15.9)		27 (26.0)	66 (15.7)	
*No vaccination*	n (%)	277 (51.3)	49 (45.0)	222 (53.5)		46 (44.2)	226 (53.8)	
*1 vaccination*	n (%)	16 (3.0)	2 (1.8)	14 (3.4)		4 (3.8)	12 (2.9)	
≥ *2 vaccinations*	n (%)	145 (26.9)	28 (25.7)	113 (27.2)		27 (26.0)	116 (27.6)	
***Beta-blocker intake***								
*7 day prior to PE*	n (%)	139 (26.1)	37 (34.3)	97 (23.7)	**0.034**	34 (33.3)	99 (23.8)	0.064
*At hospital discharge*	n (%)	183 (34.3)	42 (38.9)	134 (32.6)	0.265	42 (40.8)	134 (32.2)	0.127
*7 day prior to 3-month Follow-up*	n (%)	178 (40.3)	40 (40.8)	134 (40.1)	0.995	41 (47.1)	132 (38.3)	0.166
***Statins intake***								
*7 day prior to PE*	n (%)	98 (18.4)	29 (26.9)	66 (16.1)	**0.015**	18 (17.6)	79 (19.0)	0.865
*At hospital discharge*	n (%)	183 (34.3)	42 (38.9)	134 (32.6)	0.265	22 (21.4)	92 (22.1)	0.974
*7 day prior to 3-month Follow-up*	n (%)	118 (26.7)	31 (31.6)	84 (25.1)	0.251	24 (27.6)	93 (27.0)	1
***Antidepressants intake**** (7 days prior tp PE)*	n (%)	49 (9.2)	18 (16.7)	30 (7.3)	**0.005**	14 (13.7)	34 (8.2)	0.123

^a^The total sample also includes cases with valid data only for on exposure (depression or anxiety), so that the subgroups do not add up to the total sample size

^b^HADS Depression Score ≥ 8

^c^HADS Anxiety Score ≥ 8

^d^*HADS* Hospital Anxiety and Depression Scale

^e^Computed tomography of the lungs with intravenous contrast agent (Pulmonary CT)

^f^*BMI* Body mass index

^g^*PE* Pulmonary embolism

^h^Chronic conditions are defined as either chronic kidney disease or diabetes, or both

^i^*EQ VAS* EuroQol Visual Analogue Scale

^j^*SARS-CoV-2* Severe acute respiratory syndrome coronavirus 2

^*^SD Standard deviation

^**^IQR Interquartile range

Participants with depressive symptoms were significantly more likely to use beta-blockers, statins, and antidepressants 7 day prior to PE compared to those without depression. Frequency of medication with beta-blockers and statins increased at hospital discharge and even more so at three-month follow-up compared to 7 days prior to PE. However, there were no significant differences in medication between patients with depression or anxiety at the two later time points. Additionally, a prior diagnosis of depression, the presence of chronic conditions, and lower levels of education were more common in the depressive group, which also exhibited significantly lower subjective health status (EQ VAS). No group differences were observed regarding the severity of the PE event (bilateral PE, central thrombus components, infiltrates). Similarly, participants with symptoms of anxiety were more likely to have a prior diagnosis of depression, chronic conditions, and lower levels of education. They also exhibited significantly lower EQ VAS scores compared to those without anxiety.

Of the initial cohort of 811 participants from the LEA study, a total of 273 participants were excluded from the analysis due to missing response to the 3-month follow-up survey. The characteristics of the excluded cohort can be found in supplementary Table S1. In comparison to the excluded study population, participants included in the analysis were non-significantly younger, had a significantly lower prevalence of chronic conditions and cancer, and were less likely to have a history of depression. Additionally, the use of beta-blockers and antidepressants prior to the PE event was less common in the included group.

### Medication exposure and mental health after PE

The prevalence of an abnormal depression score (HADS-D ≥ 8) at the first follow-up was 109 cases (20.3%), while an abnormal score for anxiety (HADS-A ≥ 8) was observed in 104 cases (19.3%). Sixty-two of the patients experienced high scores for both depression and anxiety at three months follow-up. Medication exposure was present in 139 cases (26.1%) for beta-blockers and 98 cases (18.4%) for statins. A total of 48 patients were using both medications at baseline.

### Association of beta-blockers and statins with symptoms of depression and anxiety in PE patients

The results of the multivariable linear regression models showed a significantly positive association between the use of beta-blockers prior to PE and the HADS-D scores for depression (β = 0.87, 95% CI: 0.01–1.74, *p* = 0.046) and anxiety (β = 0.87, 95% CI: 0.05–1.69, *p* = 0.037) at follow-up after three months, see Table 2. No significant association was observed between the use of statins prior to PE and either depression or anxiety (Table 2). A sensitivity analysis was conducted to ensure the robustness of the findings and confirmed the association between beta-blockers and anxiety (β = 0.88, 95% CI: 0.08–1.68 *p* = 0.031), see Table S2 (Supplements). The second sensitivity analysis (using a combined variable for beta-blocker and statin use) confirmed the significant association between use of beta-blockers prior to PE and depression and anxiety at three-month follow-up (see supplementary Table S3). Although not reaching significance due to reduced sample sizes, the subgroup analyses (including only patients hospitalized before or after January 1, 2020) confirmed the results found for the association between beta-blockers and depression/anxiety, see supplementary table S4. In accordance with the main models, we did not find any significant associations for statins, see supplementary table S5. However, the beta coefficients were negative for the early period and positive for the latter. Finally, the models using intra-hospital HADS scores as outcome (supplementary table S6) showed no significant association either for beta-blockers or for statins, which indicates that the found associations are valid for the early phase of recovery after PE but not in the context of the acute event during the hospital stay.

**Table 2:** Results of the multivariable linear regression models analysing the association between beta-blocker or statin use prior to pulmonary embolism (PE) event and presence of depression and anxiety three months after PE

		**Outcome depression**					**Outcome anxiety**		
**Exposure**	**Adjusted R**^**2**^	**Beta**	**95% CI**^**1**^	***p*-value**	**Exposure**	**Adjusted R**^**2**^	**Beta**	**95% CI**^**1**^	***p*-value**
Model 1	0.092				Model 3	0.022			
Beta-blockers		0.87	0.01–1.74	**0.046**	Beta-blockers		0.87	0.05–1.69	**0.037**
Model 2	0.086				Model 4	0.030			
Statins		0.44	−0.53–1.41	0.378	Statins		0.10	−0.82–1.02	0.834
Observations	472				Observations	473			

Model adjusted for age, gender, prior depression, prior PE event, BMI, smoking status, medication with antidepressants and education

^1^*95% CI* 95% confidence interval

## Discussion

The results of the present analysis show a significant association between the medication with beta-blockers before PE and increased symptoms of depression and anxiety three months after the acute event. There was no significant association between statin use and depression and anxiety, respectively.

### Study sample and prevalence of depression and anxiety in PE patients

The study sample majorly consisted of older adults, who suffered from various comorbidities and cardiovascular conditions such as hypertension. In these individuals, dementia and declining cognitive abilities are quite common [21] and it can be suspected that this circumstance alone impacted mental health. As the present study indicates, approximately one in five participants experienced either relevant symptoms of anxiety or depression three months after PE. Participants excluded from this analysis due to missing follow-up data tended to be older and exhibited characteristics associated with greater vulnerability to psychological disorders. Therefore, the actual prevalence of depression and anxiety in the study population may have been underestimated.

### Beta-blockers and statins in PE patients

Beta-blockers and statins are widely used for prevention of cardiovascular complications [2, 34]. They are also commonly prescribed in PE patients, since these patients are often affected by other cardiovascular comorbidities (high blood pressure, coronary artery diseases, etc.) as well.

Likewise, statins are frequently prescribed in PE patients with concomitant cardiovascular comorbidities in order to manage and reduce the risk of associated complications. Statins lower blood cholesterol levels by inhibiting the enzyme HMG-CoA reductase, which is essential for cholesterol biosynthesis in the liver [29, 36]. Current literature indicates that statins also reduce inflammatory processes and endothelial dysfunction, which lowers the overall risk of several cardiovascular conditions [26, 36].

### Beta-blockers and mental health

The psychiatric effects of beta-blockers were extensively analyzed in previous studies with mixed findings. Some studies suggest a potential association of beta-blockers with increased depressive symptoms in myocardial infarction patients [27]. The associations were also seen in patients suffering from hypertension [43] and especially elderly hypertensive patients [1, 35], which is in line with our findings. However, it must be considered that in any observational study there is no randomization of treatment, which carries the risk of residual confounding by indication. In the present study, PE patients in worse overall health condition and with more comorbidities were probably more likely to be treated with beta-blockers and statins but are also at higher risk of mental health problems due to reduced health condition. Not in agreement with our results are those of a recent systematic review and meta-analysis, that did not find any association between medication with beta-blockers and depression [34], which was confirmed by the results of another systematic review from 2021 [37].

Beta-blockers inhibit the effects of stress hormones like adrenaline and noradrenaline on the beta-adrenoreceptors. Known and common physical side effects of beta-blockers are fatigue, nausea, and constipation [11]. Some patients also suffer from erectile dysfunction or report weight gain [11], which might partly mediate the potential association between beta-blocker use and increased symptoms of depression and anxiety found in the present study. However, there might be also direct effects of beta-blocker on mental health as these drugs are known to have neuropsychiatric effects. According to a meta-analysis by Archer et al., there was a substantial increase in the prescription of beta-blockers for patients with anxiety between 2003 and 2018 [3], but beneficial effects of beta-blocker treatment in patients suffering from anxiety remain unclear [3]. Next to anxiety, beta-blockers, might also be effectively used in other neuropsychiatric diseases like aggressive behavior, posttraumatic stress syndrome and or obsessive–compulsive disorder [8].

### Statin use and mental health

Several systematic reviews investigated the effects of statins on the development of depression and found robust evidence that statins are unlikely to cause depressive symptoms [14, 26, 28]. There are even some publications indicating that statins might potentially be a protective factor against the development of depression [14, 26, 31, 32, 41, 42]. In addition to their lipid-lowering effects, statins exhibit pleiotropic effects including a modulation of inflammatory processes [9, 13]. Beyond metabolic and immunological effects, statins may also exert direct neurobiological effects like promoting neuroplasticity [14].

Regarding anxiety, two large cohort studies suggest that statin use does not play a substantial role in anxiety [31, 42], which is in line with the results of the present study. Overall, studies on the association between statins and anxiety are very scarce and evidence remains inconclusive; further research in this area is needed. Some authors hypothesize that the cholesterol-lowering effects of statins could potentially increase vulnerability in some individuals, such as the elderly with already low cholesterol levels, and make them more susceptible to psychological disorders by further reducing serotonin levels [23].

### Limitations and strengths

This study has some important limitations to mention. The excluded patients were slightly older and were characterized by significantly higher use of medication and more depressive symptoms prior to PE, which might introduce a relevant selection bias towards healthier patients. Also, selection bias due to loss of follow-up must be considered. Both biases might have attenuated the analyzed associations, and the described effects might be underestimated. The exact duration and dosage of beta-blocker or statin use before hospitalization remains unknown. The physical and psychological impact of beta-blockers and statins may vary based on the length of use and the dosage. There was also no information on the exact type of beta-blockers and statins (e.g. lipophilic vs hydrophilic agents), which may have affected the associations. The recruitment phase of the LEA study includes the main phase of the COVID-19 pandemic, which has caused depression and anxiety especially in elderly [25, 40]. We cannot rule out the possibility that these circumstances may have also affected the associations analyzed in the present study. However, the subgroup analyses, including only patients hospitalized before or after January 1, 2020, suggest that the found associations are valid even though the pandemic coincided with the recruitment phase. Also, there were no significant differences between PE patients with and without depression/anxiety with regards to the number of SARS-CoV-2 vaccinations before hospitalization. Another limitation of the study is missing information on whether patients were provided with psychological care before PE. Due to the observational nature of this study, no causal relationships can be established between the variables analyzed. The relatively low R^2^ values of the linear regression models indicate potential unmeasured or residual confounding. Especially residual confounding by indication, which is caused by a non-randomized treatment, must be considered. Also, severity of PE might have played a role with regards to mental health after PE. Even though we had no information for example on the simplified Pulmonary Embolism Severity Index (sPESI), other variables that represent PE severity (bilateral PE, central thrombus components, infiltrates) showed no differences between the groups (depression/no depression and anxiety/no anxiety). Moreover, the LEA study is a single-center study, which may restrict the applicability of the results to other areas, where hospitalized PE patients may differ with regards to socioeconomic status, ethnicity or other important characteristics.

On the other hand, the study is characterized by several strengths. The study included a large number of well-characterized participants, which enabled the calculation of multivariable regression models adjusting for important confounders. The prospective nature of the study reduces the impact of recall bias. Furthermore, the usage of the German version of the HADS, a valid and reliable questionnaire for surveys in physical ill patients, allows comparison with other studies and ensures high methodological quality [6].

## Conclusion

In this study, we found a significant association between beta-blocker intake prior to PE and increased symptoms of depression and anxiety three months after PE, which indicates a potential adverse effect of beta-blockers use on mental health in PE patients. No significant associations were found for statin intake. The results suggest that physicians must be aware of these circumstances when treating PE patients and those with prior beta-blocker medication.

## Supplementary Information

Supplementary Material 1.

## Data Availability

The datasets generated during and/or analyzed in the current study are not publicly available due to data protection aspects but are available in an anonymized form from the corresponding author on reasonable request.
