# Supplementary material for: Association between beta-blocker and statin use and mental health in patients following pulmonary embolism: a prospective cohort study
Source: BMC Psychol. 2026 Jul 16;14:1063. doi: 10.1186/s40359-026-05187-w (PMC13377764; doi:10.1186/s40359-026-05187-w)
Supplement: Supplementary file 1 — Supplementary Material 1. [file 40359_2026_5187_MOESM1_ESM.pdf]

Supplements

Titel: Association between beta-blocker and statin use and mental health in patients following pulmonary embolism: A prospective cohort study

Daniel Sabljo, Simone Fischer, Thomas M Berghaus, Jakob Linseisen, Christa Meisinger, Timo Schmitz

Table S1: Comparison of baseline characteristics between excluded (without 3-month follow-up) and included patients.

		Patients with 3-month FU n = 538	Patients without 3-month FU n = 273	p value	N*
Age (years)	Mean (SD)	63.3 (14.5)	65.0 (16.7)	0.165	811
Sex - male	n (%)	292 (54.3)	133 (48.7)	0.155	811
Higher education	n (%)	152 (28.3)	38 (13.9)	**<0.001**	811
BMI (kg/m²)	Median, IQR	28.3 (24.9 - 32.9)	28.0 (23.9 - 32.7)	0.194	757
Smoking status	n (%)			**0.031**	798
Never smoker		278 (52.4)	125 (46.8)		
Former smoker		210 (39.5)	105 (39.3)		
Current smoker		43 (8.1)	37 (13.9)		
History of depression	n (%)	90 (16.8)	82 (30.1)	**<0.001**	809
Prior PE	n (%)	58 (10.8)	42 (15.4)	0.076	0.076
Chronic condition	n (%)	130 (24.3)	94 (34.8)	**0.002**	806
Cancer	n (%)	106 (19.7)	88 (32.2)	**<0.001**	811
EQ VAS (baseline)		61.0 (45.0 - 75.0)	50.0 (35.0 - 70.0)	**<0.001**	494
Beta-blocker intake[a]	n (%)	139 (26.1)	104 (39.0)	**<0.001**	799
Statins intake[a]	n (%)	98 (18.4)	63 (23.6)	0.104	799
Antidepressants intake[a]	n (%)	49 (9.2)	52 (19.5)	**<0.001**	799

* Number of cases without missing values
[a] Medication seven days prior to PE event.

Table S2: Results of the sensitivity analysis analysing the association of beta-blockers prior PE event and anxiety three months after pulmonary embolism (PE). HADS-A scores of 16 and higher were set to 15 in order to avoid a skewed distribution.

		Anxiety		
**Variable**	**Adjusted R^2**	**Beta**	**95% CI**	**p-value**
Model 1	0.022			
Beta-blockers		0.88	0.08-1.68	**0.031**
Observations	473			

Model adjusted for age, gender, prior depression, prior PE event, BMI, smoking status, medication with antidepressants and education.

Table S3: Results of the multivariable linear regression models to analyse the association between beta-blocker and/or statin use (coded in one variable) prior to pulmonary embolism (PE) event and the presence of depression and anxiety three months after PE

	Depression				Anxiety			
	**Adjusted R^2**	**Beta**	**95% CI**	**p-value**	**Adjusted R^2**	**Beta**	**95% CI**	**p-value**
	0.087				0.026			
No use (reference)		-	-	-		-	-	-
Only beta-blockers		1.13	0.09-2.17	**0.032***		1.07	0.08-2.06	**0.034***
Only statins		0.64	-0.68-1.97	0.338		0.29	-0.96-1.54	0.645
Beta-blockers and statins		1.06	-0.27-2.38	0.117		0.67	-0.57-1.92	0.289
Observations	472				473			

The model was adjusted for age, gender, prior depression, prior PE event, BMI, smoking status, medication with antidepressants and education.

Table S4: Results of the multivariable linear regression models only including PE cases hospitalized before January 1, 2020

	Depression (N=160)				Anxiety (N=159)		
**Exposure**	**Beta**	**95% CI**	**p-value**	**Exposure**	**Beta**	**95% CI**	**p-value**
Beta-blockers	0.94	-0.51-2.39	0.202	Beta-blockers	1.35	-0.05-2.75	0.059
Statins	-0.36	-2.08-1.36	0.679	Statins	-0.81	-2.49-0.87	0.344

Model adjusted for age, gender, prior depression, prior PE event, BMI, smoking status, medication with antidepressants and education.

Table S5: Results of the multivariable linear regression models only including PE cases, that were hospitalized from January 1, 2020.

	Depression (N=282)				Anxiety (N=284)		
**Exposure**	**Beta**	**95% CI**	**p-value**	**Exposure**	**Beta**	**95% CI**	**p-value**
Beta-blockers	1.13	0.02-2.24	**0.045**	Beta-blockers	0.72	-0.34-1.79	0.180
Statins	0.39	-0.86-1.65	0.538	Statins	0.35	-0.83-1.54	0.559

Model adjusted for age, gender, prior depression, prior PE event, BMI, smoking status, medication with antidepressants and education.

Table S6: Results of the multivariable linear regression models using HADS Depression Score and HADS Anxiety Score at baseline (data collected during hospital stay) as outcome.

	**Depression (N=430)**				**Anxiety (N=428)**		
**Exposure**	**Beta**	**95% CI**	**p-value**	**Exposure**	**Beta**	**95% CI**	**p-value**
Beta-blockers	0.23	-0.75-1.24	0.639	Beta-blockers	-0.39	-1.32-0.54	0.410
Statins	0.23	-0.90-1.37	0.686	Statins	0.53	-0.53-1.59	0.326

Model adjusted for age, gender, prior depression, prior PE event, BMI, smoking status, medication with antidepressants and education.

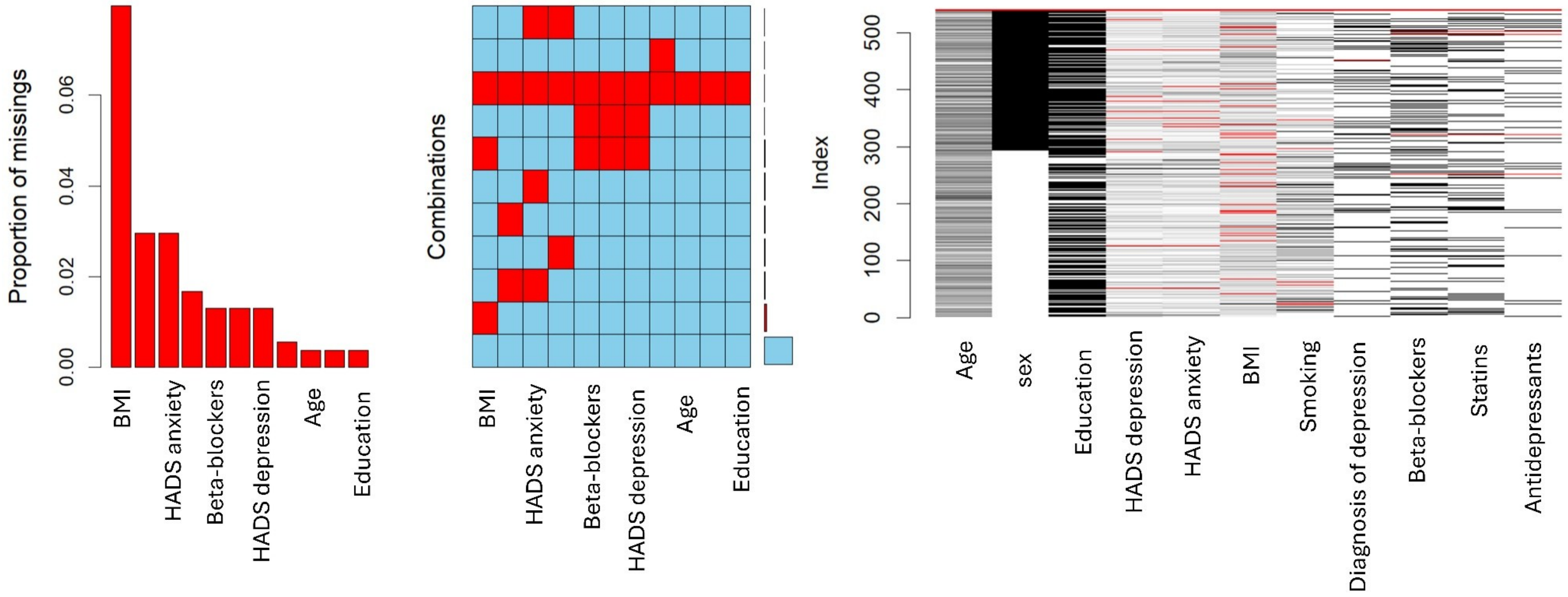

Figure S1: Missing value analyses of variable included into regression models.
